# Home for Incurables in Chicago

**Published:** 1885-03

**Authors:** 


HOME FOR INCURABLES IN CHICAGO.

The will of the late Clarissa C. Peck (widow of Philander Peck), who died Dec. 22, 1884, was admitted to probate on the 7th inst. The Peck estate is a very wealthy one, and Mrs. Peck's share amounts to nearly $1,000,000. The chief bequest of the will is that of clause 34, which provides for a home for incurables in this city. The bequest of all the property of the testatrix, not otherwise bequeathed, is to this noble purpose. The exact amount of the bequest is not definitely known, but it will exceed $600,000. The sum of money to be expended in the erection of suitable buildings, in furnishing the same, and in fitting up the grounds, is limited to $125,-000. The site of the home will be in the city of Chicago, or township of Hyde Park, Cook county. A corporate organization is directed to be founded under the statutes of the State of Illinois within two years after the death of the testatrix. Among the trustees and corporators of the corporation occurs the name of Charles Gilman Smith, M. D.

The testatrix alludes to the scope of the institution and the functions of the trustees in the following terms: "While I wish said institution to be open to all incurables without distinction up to the limits of its means and capacity, yet I direct that its Board of Trustees shall have full power to exercise their discretion in regard to the admission of patients, and also to the terms and conditions under which patients shall share in the benefits of said institution, and that said Board of Trustees shall have full and unrestricted power and control in all respects, subject only to the limitation that they shall endeavor to carry out the general policy herein indicated, and accomplish the greatest good possible for poor incurables with the means placed at their disposal."

---

The Illinois State Board of Health will hold its next meeting at the Grand Pacific Hotel in Chicago, on Thursday, April 16th prox. This is the meeting at which the annual examination of candidates for certificates is held, and such candidates are notified that the examination will cover the subject of preliminary education.

